# Acupuncture and related therapies for the anxiety and depression in irritable bowel syndrome with diarrhea (IBS-D): A network meta-analysis of randomized controlled trials

**DOI:** 10.3389/fpsyt.2022.1067329

**Published:** 2022-12-23

**Authors:** Xuesong Wang, Xuliang Shi, Jing Lv, Juncha Zhang, Yongli Huo, Guang Zuo, Guangtong Lu, Cunzhi Liu, Yanfen She

**Affiliations:** ^1^School of Acupuncture-Moxibustion and Tuina, Hebei University of Chinese Medicine, Shijiazhuang, Hebei, China; ^2^Hebei International Joint Research Center for Dominant Diseases in Chinese Medicine and Acupuncture, Hebei University of Chinese Medicine, Shijiazhuang, Hebei, China; ^3^Department of Spleen and Stomach, Hebei Province Hospital of Chinese Medicine, Shijiazhuang, Hebei, China; ^4^School of Acupuncture-Moxibustion and Tuina, Beijing University of Chinese Medicine, Beijing, China

**Keywords:** acupuncture, irritable bowel syndrome with diarrhea, anxiety, depression, network meta-analysis, randomized controlled trials

## Abstract

**Objective:**

A growing number of clinical studies have suggested the value of acupuncture-related therapies for patients with irritable bowel syndrome with diarrhea (IBS-D), and the patient’s mental state plays an important role, but there are many types of acupuncture-related therapies involved. This study aimed to evaluate the mental status, efficacy and safety of the different acupuncture-related therapies for IBS-D patients.

**Methods:**

We searched seven databases to collect randomized controlled trials of acupuncture-related therapies for IBS-D. After independent literature screening and data extraction, the quality of the final included literature was evaluated. Hamilton anxiety rating scale (HAMA), hamilton depression rating scale (HAMD), self-rating anxiety scale (SAS), and self-rating depression scale (SDS) was used as the primary outcome indicator. And the network meta-analysis (NMA) was performed by using Revman 5.4, Stata 15.0 and WinBUGS 1.4.3 software, and the surface under the cumulative ranking curve was conducted to rank the included interventions.

**Results:**

We analyzed 24 eligible studies with 1,885 patients, involving eight types of acupuncture and related therapies along with comprehensive therapies. The NMA result shows that: for SAS scores, combined therapies were more efficacious than anti-diarrheal or antispasmodic (western medicine, WM) (SMD: −8.92; 95% CI: −15.30, −2.47); for SDS scores, combined therapies were more efficacious than WM (SMD: −8.45; 95% CI: −15.50, −1.41). For HAMA scores, moxibustion (MOX) was more efficacious than placebo (SMD: −8.66; 95% CI: −16.64, −0.38). For HAMD scores, MOX was more efficacious than all other included interventions. For response rate, MOX was more efficacious than the following interventions: acupuncture (ACU) (SMD:0.29; 95% CI:0.08,0.93), Chinese herb medicine (CH) (SMD:0.09; 95% CI:0.02,0.36), combined therapies (SMD:0.23; 95% CI:0.06, 0.85), electroacupuncture (EA) (SMD:0.06; 95% CI:0.01,0.33), warm acupuncture (WA) (SMD:22.16; 95% CI:3.53,148.10), WM (SMD:15.59; 95% CI:4.68,61.21), and placebo (SMD:9.80; 95% CI:2.90,45.51). Combined therapies were more efficacious than the following interventions: CH (SMD:0.39; 95% CI:0.19,0.80), WA (SMD:4.96; 95% CI:1.30,21.62), and WM (SMD:3.62; 95% CI:2.35,5.66). The comprehensive ranking results show that MOX, ACU, combined therapies, and EA had high SUCRA rankings involving different outcome indicators.

**Conclusion:**

MOX, ACU, combined therapies, and EA better alleviate anxiety and depression among IBS-D patients, and with a higher safety level, may be the optimal therapies. In addition, combining acupuncture-related treatments and other therapies also delivers a higher global benefit level.

**Systematic review registration:**

[https://www.crd.york.ac.uk/], identifier [CRD42022364560].

## 1 Background

Abdominal pain, inconsistent bowel movements, and altered stool characteristics are the hallmarks of irritable bowel syndrome (IBS), a functional bowel illness (1, 2). The prevalence varies from country to country, with an overall global prevalence of approximately 15% (3), with a 19.58–23.40% rate in China and 10–25% (4) in North America and Europe (5). The prevalence also is higher in females than in males (6–8). Based on the clinical symptoms, irritable bowel syndrome with diarrhoeal (IBS-D) is the most common, in addition to irritable bowel syndrome with predominant constipation (IBS-C), irritable bowel syndrome with mixed bowel habits (IBS-M), and irritable bowel syndrome unclassified (IBS-U), four types in total (3). Symptomatic medications, such as antispasmodics, antidiarrheal, prokinetic, and laxatives, are the ones most frequently used to treat IBS. Despite the fact that these medications partially alleviate IBS symptoms, prolonged use might lead to adverse effects such as ischemic colitis (9).

Patients often suffer from a heavy burden of anxiety and depression due to the long course and recurrent episodes of IBS, with a high prevalence of anxiety (31.4%) and depression (37.1%) in IBS patients (10). Similarly, the previous systematic review also indicated that IBS patients had significantly higher levels of anxiety and depression, which could further aggravate the symptoms (11). The relationship between the psychological factors and IBS is also characterized by the latest Rome IV criteria (12). Therefore, the status of anxiety, depression, and other psychological status in IBS treatment cannot be ignored. During the treatment process, we should focus not only on improving the symptoms but also on the changes in the patient’s mental state. However, because psychological issues are complicated, it might be challenging to treat a wide range of symptoms with a single drug. Antipsychotics that are frequently prescribed in clinics might help with the symptoms, but prolonged usage has a risk of negative consequences (such as drug habit and resistance) (13).

Given the inadequacy of pharmacological therapy, several patients have resorted to acupuncture as an additional and alternative treatment. As an essential part of traditional Chinese medicine, acupuncture has a long history of treating gastrointestinal disorders. Compared with pharmacological therapy, acupuncture therapy has less side effects. Plenty of clinical studies have shown the effectiveness and safety of acupuncture for IBS (14–16), and recent evidence-based research has also confirmed this view (17, 18). Furthermore, the effects of acupuncture are comprehensive in that they can relieve the IBS symptoms and benefit patients’ psychological conditions (19). Previous studies have typically compared different acupuncture-related therapies as a whole with other interventions, which focused on pairwise comparisons. Despite the wide use of acupuncture, the current evidence on acupuncture appears to be conflicting. Different studies showed different results. One systematic review showed that acupuncture and sham acupuncture were similarly effective in improving IBS symptoms (20), while another meta-analysis showed that acupuncture was more effective than pharmacological therapies (21). Adding to the fact that acupuncture-related therapies include various types, such as acupuncture, electroacupuncture, moxibustion, it could make us more confused. Therefore, we used a network meta-analysis (NMA) to compare and rank the different types of acupuncture-related therapies for IBS-D patients’ anxiety and depression status.

## 2 Methods

Our study was conducted based on the checklist of the preferred reporting items for systematic reviews and meta-analyses for network meta-analysis (PRISMA-NMA) guidelines (Supplementary Appendix 1) (22) and reporting items of systematic reviews and meta-analyses involving acupuncture (23). The PRISMA- NMA and PRISMA for acupuncture checklists are both updates to PRISMA. Based on the 27 items from the PRISMA checklist, the PRISMA-NMA checklist revised 11 items related to NMA and added 5 new items to guide and improve the writing and reporting for NMA, while PRISMA for acupuncture checklist revised 6 items related to acupuncture operations and added 5 new items to be better used for the systematic review about acupuncture therapies. This study has been registered in PROSPERO,^1^ and the registration number was CRD42022364560 (24).

### 2.1 Search strategies

To access randomized controlled trials (RCTs) of acupuncture-related therapies for IBS-D, we searched three English databases (PubMed, EMBASE, Cochrane Library) and four Chinese databases [China Biology Medicine (CBM), China National Knowledge Infrastructure (CNKI), Wanfang Data, and Chinese Scientific Journal Database (VIP)] from their inception to January 15, 2022. Medical Subject Headings (MeSH) terms and free words were used in the search (Supplementary Appendix 2).

### 2.2 Inclusion criteria

(1) Published literature, where the type of literature was Randomized Controlled Trials (RCT), with no restrictions on patient age or gender. (2) Accurate diagnostic criteria for IBS-D have been reported in the literature, such as Rome I-IV (25–28), or expert consensus (29). (3) The patient was accompanied by an anxious and/or depressed state, and had a clear diagnosis. (4) The interventions in the treatment group were acupuncture (ACU), electro-acupuncture (EA), warm acupuncture (WA), moxibustion (MOX), and other acupuncture-related therapies (such as the combination of acupuncture and medication), while the control group was anti-diarrheal or antispasmodic (western medicine, WM), placebo, or the comparison of various acupuncture-related therapies. In order to include acupuncture-related therapies more comprehensively, any group of studies that used acupuncture-related therapies were retained. (5) The only available publication languages were Chinese or English.

### 2.3 Exclusion criteria

(1) Acupuncture-related therapies were used for intervention, but no indicators of anxiety and/or depression were reported; (2) Other types of IBS studies (such as IBS-C, IBS-M, IBS-U, among others); (3) The same group included three and more interventions; (4) Research with no clear original data reported and the author was not reachable; (5) similar research or reported the same results.

### 2.4 Primary and secondary outcomes

At least one primary outcome indicator was reported: Hamilton anxiety rating scale (HAMA), hamilton depression rating scale (HAMD), self-rating anxiety scale (SAS), self-rating depression scale (SDS).

Secondary outcomes: Response rate: the proportion of patients who achieved satisfactory improvement in global symptoms. For studies classified as *“apparently effective*,” *“effective*,” and *“ineffective*,” we labeled *“apparently effective”* and *“effective”* as *“improvement*,” and *“ineffective”* was marked as *“no improvement”*; for studies classified as *“clinically cured,” “apparently effective,” “effective,” “ineffective,”* we labeled *“clinically cured,” “apparently effective,” “effective”* as *“improvement,”* and *“ineffective”* was marked as *“no improvement”*.

### 2.5 Literature screening and data extraction

Two researchers independently screened the literature and extracted data, cross-checking the results obtained and inviting the third researcher to adjudicate in case of disagreement. For data extraction, we used previously prepared tables that contained the following information: (1) General Information: the name of the first author, the name of the journal, group information, number of patients in each group, etc.; (2) Baseline data: age and course of patients in each group, before treatment data for each outcome indicator; (3) Interventions: type of acupuncture, frequency and period of treatment; (4) post-treatment data for each outcome indicator, and adverse effects; (5) risk bias-related factors: random sequence generation, allocation, blinding, etc.

### 2.6 Risk assessment of bias in inclusion studies

Our two researchers independently evaluated the risk bias of the included studies, using the risk of bias assessment tool recommended by the Cochrane Handbook 5.1 (30, 31).

### 2.7 Statistical analysis

Software programs RevMan (Version 5.4), Stata (Version 15.0), and WinBUGS (Version 1.4.3) were used to do statistical analysis (32, 33). SAS, SDS, HAMA, and HDMA scores were numerical variables, and standard mean difference (SMD) was used; the response rate was categorically variable, and risk ratio (RR) was used.

First, the pairwise meta-analysis was performed using RevMan manager. The *I*-square (*I*^2^) test was performed to determine the degree of heterogeneity among the RCTs (34). They were examined using the random-effects model if *I*^2^ was greater than 50%; if not, a fixed-effect model was utilized (35). Subgroup analyses were performed according to the different interventions, using the 95% confidence interval (95% CI) of SMD and RR as effect sizes to indicate the relative strength of efficacy.

Second, Stata15.0 was employed to draw an NMA evidence relationship diagram. The study was divided and restructured into all-paired two-arm trials if it had been a three-arm trial (36).

Next, The WinBugs 1.43 software will be run to perform a network meta-analysis of the data, using Markov Chain-Monte Carlo (MCMC) for Bayesian inference to infer the posterior probabilities based on the prior probabilities, and estimation and inference under the assumption that MCMC has reached a steady state of convergence. When running the WinBUGS program, the number of iterations is set to 1,00,000, and the first 10 000 are used for annealing to eliminate the effect of the initial values. When there is a closed loop, the direct evidence and indirect evidence are consistent if the 95% CI of the inconsistency factors (IF) contains 0, otherwise, there is a possibility of inconsistency being run (37).

Next, funnel plots were produced using Stata 15.0 to assess the presence of minor sample effects within the included studies (38).

Finally, the surface under the cumulative ranking curve (SUCRA) was generated with Stata 15.0, which shows the SUCRA scores for all interventions, with higher SUCRA values denoting a higher treatment class (39).

## 3 Results

### 3.1 Study search and description

**FIGURE 1:**
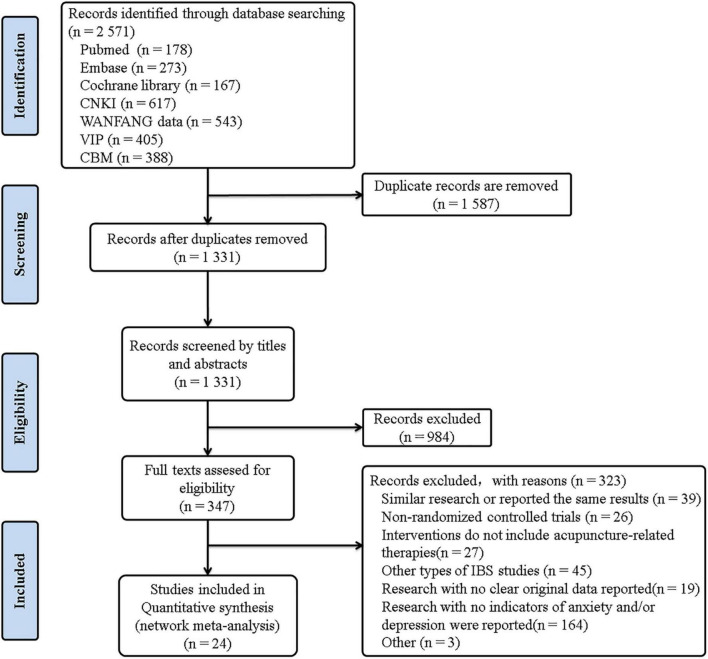
Study flow diagram.

The literature search turned up a total of 2,571 pertinent studies. The final screening contained 24 RCTs with 1,885 IBS-D patients, 23 of which were in Chinese. In Figure 1, the findings of the literature screening process are displayed. Eight interventions were included: acupuncture (ACU), moxibustion (MOX), electroacupuncture (EA), Chinese herb medicine (CH), warm acupuncture (WA), the combined acupuncture-related therapies and other therapies (combined therapies), anti-diarrheal or antispasmodic (western medicine, WM), and placebo acupuncture/blank control (placebo). Three of them were three-armed trials (40–42), and the remaining were all two-armed trials. The baseline characteristics and the detailed acupuncture methods of the included studies are shown in Tables 1, 2.

**TABLE 1:** Baseline characteristics of the included studies.

References	Country	Diagnostic criteria	Intervention			Control			Course	Type of outcomes
			**Treatment**	***n***	**Years (Mean ± sd)/Range**	**Treatment**	***n***	**Years (Mean ± sd)/Range**		
Bu and Lv (55)	China	Rome III	Combined therapies	46	20–49 (34.2 ± 5.2)	WM	46	21–47 (34.1 ± 5.2)	10 W	➀➁➄
Chen et al. (56)	China	Rome IV	Combined therapies	31	41 ± 6	WM	30	39 ± 7	6 W	➂➃➄
Chen et al. (57)	China	Rome III	EA	34	24–62 (41.90 ± 10.01)	WM	30	25–56 (40.50 ± 8.75)	4 W	➂➃➄
Han and Feng (58)	China	Rome III	Combined therapies	144	41 ± 11.12	WM	72	40 ± 10.72	8 W	➀➄
Han et al. (59)	China	Rome III	Combined therapies	40	42.05 ± 8.87	CH	40	41.72 ± 8.03	4 W	➀➁➄
Huang et al. (60)	China	Rome III	MOX	29	30.75 ± 8.79	ACU	28	31.42 ± 9.35	4 W	➂➃➄
Li et al. (61)	China	Rome III	MOX	30	18–65 (44.27 ± 11.95)	WM	30	18–65 (44.17 ± 13.78)	4 W	➀➁➄
Li et al. (62)	China	Rome IV	Combined therapies	47	37 ± 9	WM	46	40 ± 9	2 W	➀➁➄
Meng (63)	China	Rome IV	ACU	35	39.3 ± 11.5	WM	35	38.4 ± 13.5	4 W	➁➄
Shu (64)	China	Expert consensus	WA	30	23–62 (43.26 ± 12.08)	WM	25	23–62 (43.26 ± 12.08)	1 M	➀➁➄
Sun et al. (65)	China	Rome IV	ACU	36	20–58(39 ± 10)	EA	37	21–60 (41 ± 10)	4 W	➃➄
Tian et al. (66)	China	Rome III	ACU	43	47–80 (59 ± 2.1)	Placebo	43	51–83(64 ± 1.3)	3 W	➂➃➄
Wang et al. (40)	China	Rome IV	Combined therapies	41	25–41 (29 ± 5)	WM	41	24–40 (29 ± 6)	4 W	➀➁➄
			CH	41	24–40 (29 ± 6)					
Yang et al. (41)	China	Rome III	ACU	20	55.0 ± 5.4	WM	20	54.0 ± 6.1	2 W	➂➃➄
			Combined therapies	20	54.0 ± 6.3					
Zhong et al. (67)	China	Rome III	EA	30	31.64 ± 12.31	WM	30	30.22 ± 13.99	4 W	➀➁
Zhou et al. (68)	China	Rome III	Combined therapies	45	16–64	WM	45	17–65	8 W	➂➃➄
Zhong et al. (69)	China	Rome III	EA	30	30.6 ± 13.35	WM	21	30.22 ± 13.99	4 W	➀➁
Chen (70)	China	Rome IV	Combined therapies	34	40.89 ± 10.56	WM	34	40.10 ± 10.00	4 W	➂➃➄
Li (71)	China	Rome III	EA	35	39.1 ± 11.8	WM	35	37.93 ± 11.45	4 W	➀➁
Li (72)	China	Rome III	ACU	76	23–70 (46.3 ± 13.2)	WM	33	23–65 (48.9 ± 12.4)	6 W	➀➁➄
Liang (73)	China	Rome III	ACU	22	46.45 ± 11.35	WM	12	50.83 ± 14.23	6 W	➃➄
Liu (74)	China	Rome III	MOX	30	36.50 ± 13.69	Placebo	30	35.47 ± 11.55	4 W	➀➁➄
Xiao (42)	China	Rome IV	ACU	29	43.86 ± 10.36	WM	29	40.51 ± 9.95	4 W	➃➄
			CH	30	39.37 ± 9.67					
Yang (75)	China	Rome IV	ACU	33	41.52 ± 9.10	WM	32	42.22 ± 8.71	4 W	➂➃

ACU, acupuncture; EA, electroacupuncture; MOX, moxibustion; CH, Chinese herb medicine; WA, warm acupuncture; combined therapies, the combination of acupuncture-related treatments and other therapies; WM, western medicine of anti-diarrheal or antispasmodic; placebo, and placebo acupuncture/blank control; NR, No reported; W, week; M, month; ➀, SAS; ➁, SDS; ➂, HAMA; ➃, HAMD; ➄, Response rate.

**TABLE 2:** Descriptions of the included acupuncture and related therapies.

References	Style of acupuncture	Names of acupuncture points used	Retention time	Acupuncturist qualifications	Acupuncture reaction	Frequency and course of acupuncture
Bu and Lv (55)	Combined therapies	ACU + WM:Sanyinjiao (SP6), Tianshu (ST25), Zusanli (ST36), Taichong (LR3), Zhongwan (RN12), Shangjuxu (ST37), Baihui (DU20)	30 min	NR	*Deqi*	Once a day for 10 weeks
Chen et al. (56)	Combined therapies	EA + MOX:Sishencong (EX-HN1), Shenque (RN8), Tianshu (ST25), Shuidao (ST28), Shangjuxu (ST37), Yinlingquan (SP9), Taichong (LR3), Fuliu (KI7), Neiguan (PC6)	25 min	NR	*Deqi*	Once a day, 5 times a week for 6 weeks
Chen et al. (57)	EA	Baihui (DU20), Shenting (DU24), Neiguan (PC6), Shenmen (HT7), Zhongwan (RN12), Tianshu (ST25), Qihai (RN6), Sanyinjiao (SP6), Taichong (LR3)	30 min	NR	*Deqi*	Once a day, 6 times a week for 4 weeks
Han and Feng (58)	Combined therapies	EA + CH: Tianshu (ST25), Zusanli (ST36), Pishu (BL20), Weishu (BL21), Shenshu (BL23), Dachangshu (BL25), Shangjuxu (ST37), Yintang (EX-HN3), Neiguan (PC6), Taichong (LR3), Sishencong (EX-HN1)	20 min	NR	*Deqi*	Once a day for 8 weeks
Han et al. (59)	Combined therapies	EA + CH: Shangjuxu (ST37), Tianshu (ST25), Zusanli (ST36), Taichong (LR3), Yintang (EX-HN3), Baihui (DU20), Sanyinjiao (SP6)	30 min	NR	*Deqi*	Once a day for 4 weeks
Huang et al. (60)	MOX	Bladder meridian, Governor Vessel	NR	NR	NR	Once a week for 4 weeks
	ACU	Zusanli (ST36), Tianshu (ST25), Guanyuan (RN4), Zhongwan (RN12), Dachangshu (BL25), Pishu (BL20)	30 min	NR	NR	Three times a week for 4 weeks
Li et al. (61)	MOX	Shenque (RN8)	1 h	NR	NR	Once a day, 5 times a week for 4 weeks
Li et al. (62)	Combined therapies	ACU + MOX: Neiguan (PC6), Tianshu (ST25), Zusanli (ST36), Shangjuxu (ST37), Sanyinjiao (SP6), Taichong (LR3),Yintang (EX-HN3)穴, Shenque (RN8)	30 min	NR	*Deqi*	Once a day, 5 times a week for 2 weeks
Meng (63)	ACU	Taichong (LR 3), Zusanli (ST 36), Shangjuxu (ST 37), Sanyinjiao (SP 6), Tianshu (ST 25), Baihui (GV 20) and Yintang (GV 29)	30 min	NR	*Deqi*	Once a day, 5 times a week for 4 weeks
Shu (64)	WA	Pishu (BL20), Tianshu (ST25), Zusanli (ST36), Taichong (LR3), Sanyinjiao (SP6)	30 min	NR	*Deqi*	Twice a week for 1 month
Sun et al. (65)	ACU	Baihui (DU20), Shenting (DU24), Benshen (BG13)	30 min	NR	NR	Once a day, 6 times a week for 4 weeks
	EA	Guanyuan (RN4), Zhongwan (RN12), Tianshu (ST25), Dachangshu (BL25), Zusanli (ST36), Shangjuxu (ST37), Hegu (LI4), Taichong (LR3)	30 min	NR	NR	Once a day, 6 times a week for 4 weeks
Tian et al. (66)	ACU	Shangwan (RN13), Zhongwan (RN12), Xiawan (RN10), Qihai (RN6), Tianshu (ST25), Neiguan (PC6), Zusanli (ST36)	NR	NR	*Deqi*	Once a day for 3 weeks
Wang et al. (40)	Combined therapies	ACU + CH:Shangjuxu (ST37), Tianshu (ST25), Taichong (LR3), Sanyinjiao (SP6), Zusanli (ST36)	30 min	Reported	*Deqi*	Once a day, 5 times a week for 4 weeks
Yang et al. (41)	ACU	Neiguan (PC6), Tianshu (ST25), Sanyinjiao (SP6), Zusanli (ST36), Shangjuxu (ST37), Taichong (LR3), Yintang (EX-HN3)	30 min	NR	*Deqi*	Once a day, 5 times a week for 2 weeks
	Combined therapies	ACU + MOX: Neiguan (PC6), Tianshu (ST25), Sanyinjiao (SP6), Zusanli (ST36), Shangjuxu (ST37), Taichong (LR3), Yintang (EX-HN3)穴, Shenque (RN8)	30 min	NR	*Deqi*	Once a day, 5 times a week for 2 weeks
Zhong et al. (67)	EA	Tianshu (ST25), Dachangshu (BL25), Qvchi (LI11), Shangjuxu (ST37)	30 min	NR	*Deqi*	3–5 times a week for 4 weeks
Zhou et al. (68)	Combined therapies	WA + CH:Zusanli (ST36), Tianshu (ST25)	NR	NR	*NR*	Every other day for 8 weeks
Zhong et al. (69)	EA	Qvchi (LI11), Shangjuxu (ST37)	30 min	NR	*Deqi*	3–5 times a week for 4 weeks
Chen (70)	Combined therapies	ACU + MOX:Tianshu (ST25), Zusanli (ST36), Shangjuxu (ST37), Yinlingquan (SP9), Taichong (LR3), Fuliu (KI7), Neiguan (PC6), Shenmen (HT7), Baihui (DU20), Sishencong (EX-HN1), Shenque (RN8)	25 min	Reported	*Deqi*	Once a day, 5 times a week for 4 weeks
Li (71)	EA	Tianshu (ST25), Zusanli (ST36), Shangjuxu (ST37), Sanyinjiao (SP6), Taichong (LR3), Baihui (DU20), Yintang (EX-HN3)	NR	NR	*Deqi*	Once a day, 3–4 times a week for 4 weeks
Li (72)	ACU	Baihui (DU20), Yintang (EX-HN3), Tianshu (ST25), Zusanli (ST36), Shangjuxu (ST37), Sanyinjiao (SP6), Taichong (LR3)	30 min	Reported	*Deqi*	3 times a week for 6 weeks
Liang (73)	ACU	Baihui (DU20), Yintang (EX-HN3),Taichong (LR3), Zusanli (ST36), Sanyinjiao (SP6), Tianshu (ST25), Shangjuxu (ST37)	30 min	NR	*Deqi*	3 times a week for 6 weeks
Liu (74)	MOX	Shenque (RN8)	2 h	NR	NR	Once a week for 4 weeks
Xiao (42)	ACU	Feishu (BL13), Jueyinshu (BL14), Xinshu (BL15), Ganshu (BL18), Pishu (BL20), Shenshu (BL23), Chongyang (ST42), Taibai (SP3)	30 min	NR	*Deqi*	Once a week for 4 weeks
Yang (75)	ACU	Shenque (RN8)(RN8), Guanyuan (RN4)(RN4)	30 min	NR	NR	Once a day, 5 times a week for 4 weeks

ACU, acupuncture; EA, electroacupuncture; MOX, moxibustion; CH, Chinese herb medicine; WA, warm acupuncture; combined therapies, the combination of acupuncture-related therapies and other therapies; WM, western medicine of anti-diarrheal or anti-spasmodic; placebo, and placebo acupuncture/blank control; NR, No reported.

### 3.2 Quality assessment of included studies

**FIGURE 2:**
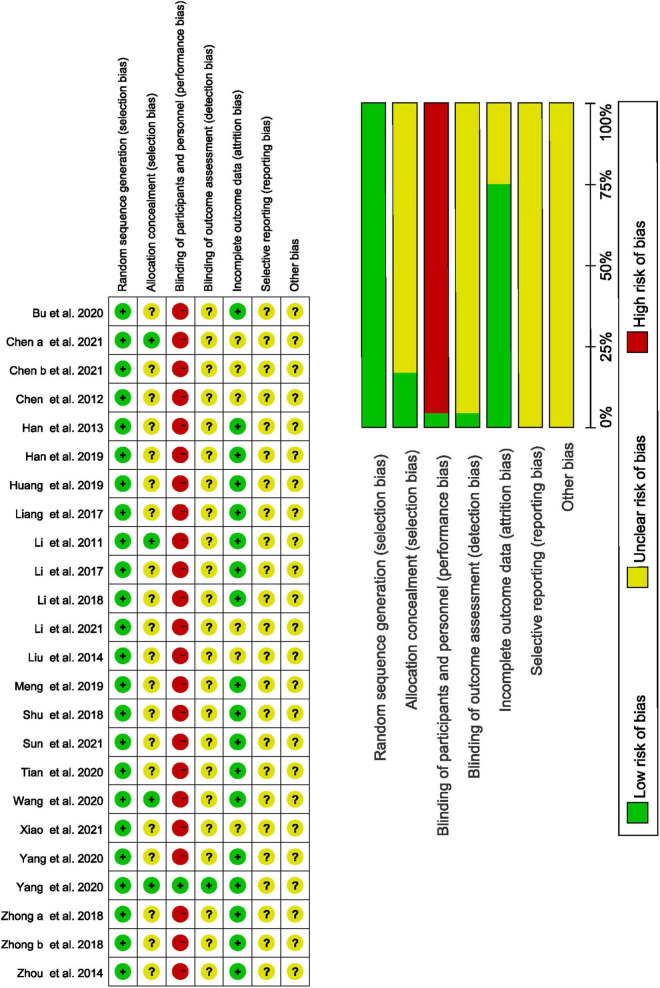
Quality assessment of included studies.

Our two researchers (GZ and GL) independently evaluated the risk bias of the included studies, includes 7 items, such as random method, allocation hiding, blind method, etc. Among the 24 trials, 24 (100%) represented a random sequence generation process, four (16.67%) described allocation concealment methods, and one (4.17%) described the blinding methods for participants or outcome assessment. Eighteen trials (75%) reported complete outcome data. Unfortunately, it is impossible to be blinded to the participants and patients because of the particularity of the acupuncture operation. The results of the risk evaluation are shown in Figure 2.

### 3.3 Results of pairwise meta-analyses

#### 3.3.1 Primary outcome: SAS, SDS, HAMA, and HAMD scores

For SAS results, combined therapies (SMD: −8.321,95% CI: −13.599, −3.043) and EA (SMD: −4.033, 95% CI: −6.643, −1.423) were more efficacious than WM. Regarding SDS, ACU (SMD: −9.455, 95% CI: −17.207, −1.702), combined therapies (SMD: −7.739, 95% CI: −11.306, −4.171), and EA (SMD: −2.651, 95% CI: −4.562, −0.739) were more efficacious than WM (Table 3). For HAMA, combined therapies (SMD: −3.478, 95% CI: −4.684, −2.273) were more efficacious than the WM. Regarding HAMD, ACU (SMD: −4.588, 95% CI: −5.996, −3.179), and combined therapies (SMD: −4.573, 95% CI: −8.735, −0.411) were more efficacious than WM (Table 3).

**TABLE 3:** Pairwise meta-analyses.

Comparison	Pairwise RR/MD (95% CI)	Number of patients	Number of studies	Heterogeneity test	
				***I*** **^2^ (%)**	***p*** ** value**
**SAS**					
Combined therapies vs WM	−8.321 [−13.599, −3.043]	483	4	92.80%	0.002*
EA vs WM	−4.033 [−6.643, −1.423]	181	3	19.10%	0.002*
**SDS**					
ACU vs WM	−9.455 [−17.207, −1.702]	179	2	51.30%	0.017*
Combined therapies vs WM	−7.739 [−11.306, −4.171]	267	3	80.50%	<0.001*
EA vs WM	−2.651 [−4.562, −0.739]	181	3	39.20%	0.007*
**HAMA**					
Combined therapies vs WM	−3.478 [−4.684, −2.273]	259	4	0.00%	<0.001*
ACU vs WM	−1.492 [−3.596, 0.612]	105	2	0.00%	0.164
**HAMD**					
ACU vs WM	−4.588 [−5.996, −3.179]	132	3	41.40%	<0.001*
Combined therapies vs WM	−4.573 [−8.735, −0.411]	194	3	86.70%	0.031*
**Response rate**					
Combined therapies vs WM	1.281 [1.182, 1.390]	742	8	0.00%	<0.001*
ACU vs WM	1.364 [1.193, 1.560]	311	5	0.00%	<0.001*
Combined therapies vs CH	1.197 [1.036, 1.383]	162	3	0.00%	0.015*
CH vs WM	1.175 [0.958, 1.440]	141	2	0.00%	0.121

ACU, acupuncture; EA, electroacupuncture; MOX, moxibustion; CH, Chinese herb medicine; WA, warm acupuncture; combined therapies, the combination of acupuncture-related therapies and other therapies; WM, western medicine of anti-diarrheal or anti-spasmodic; placebo, and placebo acupuncture/blank control; *Significant difference.

#### 3.3.2 Secondary outcome: Response rate

While assessing response rate, ACU (SMD: 1.364, 95% CI: 1.193, 1.560) and combined therapies (SMD: 1.281, 95% CI: 1.182,1.390) were more efficacious than WM. In addition, combined treatments (SMD: 1.197, 95% CI: 1.036, 1.383) were more efficacious than CH (Table 3).

### 3.4 Network meta-analysis results

**FIGURE 3:**
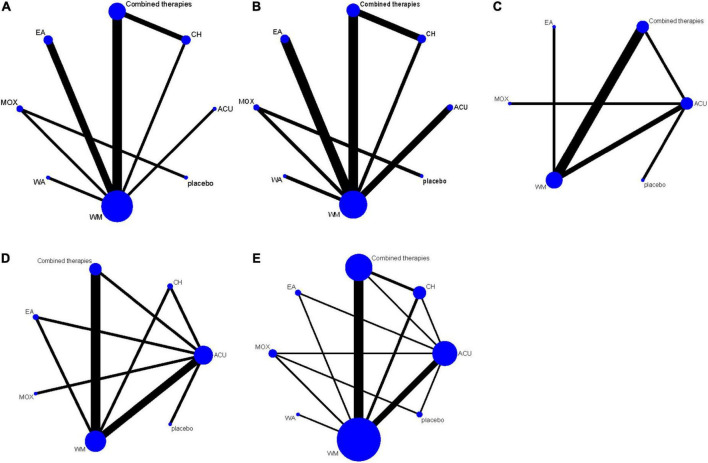
The network structure for treatment comparisons. **(A)** SAS scores; **(B)** SDS scores; **(C)** HAMA scores; **(D)** HAMD scores; **(E)** response rate. ACU, acupuncture; EA, electroacupuncture; MOX, moxibustion; CH, Chinese herb medicine; WA, warm acupuncture; combined therapies, the combination of acupuncture-related therapies and other therapies; WM, western medicine of anti-diarrheal or anti-spasmodic; placebo, and placebo acupuncture/blank control.

Five network plots were conducted using STATA 15.0. The line thickness was proportional to the two therapies, and the point size was positively correlated with the treatment sample size (Figure 3).

#### 3.4.1 Primary outcome: SAS, SDS, HAMA, and HAMD scores

**FIGURE 4:**
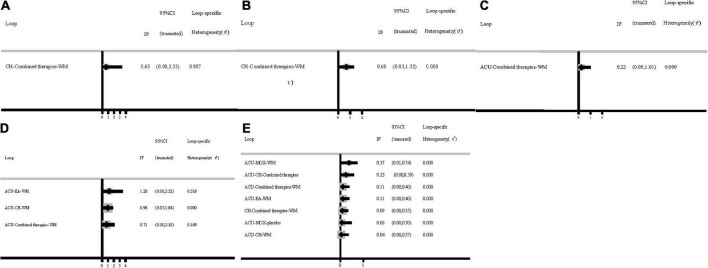
Inconsistency test. **(A)** SAS scores; **(B)** SDS scores; **(C)** HAMA scores; **(D)** HAMD scores; **(E)** response rate. ACU, acupuncture; EA, electroacupuncture; MOX, moxibustion; CH, Chinese herb medicine; WA, warm acupuncture; combined therapies, the combination of acupuncture-related therapies and other therapies; WM, western medicine of anti-diarrheal or anti-spasmodic; placebo, and placebo acupuncture/blank control.

**FIGURE 5:**
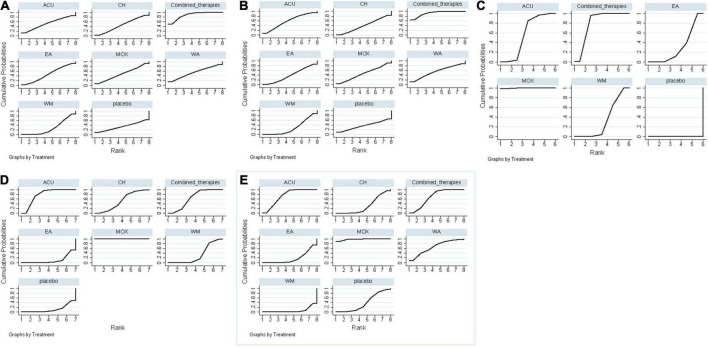
Surface under the cumulative ranking curves. **(A)** SAS scores; **(B)** SDS scores; **(C)** HAMA scores; **(D)** HAMD scores; **(E)** response rate. ACU, acupuncture; EA, electroacupuncture; MOX, moxibustion; CH, Chinese herb medicine; WA, warm acupuncture; combined therapies, the combination of acupuncture-related therapies and other therapies; WM, western medicine of anti-diarrheal or anti-spasmodic; placebo, and placebo acupuncture/blank control.

As shown in Figure 3A, 12 studies reported SAS scores involving eight interventions and 1,069 patients. Inconsistency test results show that SAS scores included one closed-loop, whose IF with 95% CI of these closed loops contained 0, indicating no obvious inconsistencies (Figure 4A). The results of the NMA showed that combined therapies were more efficacious than WM (SMD: −8.92; 95% CI: −15.30, −2.47; Table 4). The ranking results showed that the top three interventions to reduce the SAS scores were combined therapies (88.5%), WA (54.4%), and ACU (52.9%), while the worst was WM (28.3%; Figure 5A).

**TABLE 4:** Network meta-analysis results.

SAS							
**ACU**							
−5.48 (−22.89,11.99)	**CH**						
2.30 (−13.21,17.78)	7.74 (−1.74,16.55)	**Combined therapies**					
−3.86 (−20.37, 12.51)	1.51 (−11.71,14.29)	−6.19 (−16.63,4.24)	**EA**				
−0.18 (−21.93,20.90)	5.17 (−13.96,24.20)	−2.50 (−20.09,15.05)	3.61 (−14.58,21.97)	**MOX**			
1.71 (−18.89,22.22)	7.17 (−11.13,25.32)	−0.48 (−17.21,16.54)	5.75 (−11.21,22.74)	1.87 (−19.57,24.38)	**WA**		
−6.63 (−20.47,7.46)	−1.21 (−11.48,8.84)	−8.92 (−15.30, −2.47)*	−2.73 (−10.79,5.77)	−6.34 (−22.49,9.98)	−8.46 (−23.61,6.74)	**WM**	
−3.57 (−28.82,21.44)	1.88 (−22.15,25.69)	−5.77 (−28.23,16.06)	0.40 (−22.46,22.79)	−3.37 (−17.19,10.54)	−5.20 (−31.74,19.58)	3.23 (−−18.59,23.65)	**Placebo**
**SDS**							
**ACU**							
−8.44 (−22.52,5.15)	**CH**						
−1.12 (−13.62,10.77)	7.28 (−1.30,15.68)	**Combined therapies**					
−9.05 (−22.41,3.40)	−0.57 (−13.33,11.41)	−7.81 (−18.76,2.25)	**EA**				
−5.51 (−22.97,11.29)	2.98 (−13.72,19.64)	−4.29 (−19.86,11.22)	3.51 (−11.71,20.14)	**MOX**			
−1.37 (−19.39,16.02)	7.11 (−10.82,24.08)	−0.17 (−16.32,15.88)	7.71 (−8.76,24.20)	4.10 (−16.15,23.67)	**WA**		
−9.57 (−19.72,0.31)	−1.17 (−10.77,8.50)	**−8.45 (−15.50, −1.41)** *	−0.62 (−8.10,7.63)	−4.13 (−18.18,9.37)	−8.32 (−22.57,6.73)	**WM**	
−8.53 (−30.21,12.78)	−0.07 (−21.12,21.35)	−7.37 (−27.45,12.46)	0.39 (−19.17,21.30)	−3.14 (−16.62,9.96)	−7.08 (−30.83,16.52)	1.02 (−17.77,20.06)	**Placebo**
**HAMA**							
**ACU**							
0.57 (−4.17, 5.51)	**Combined_therapies**						
−2.12 (−10.59, 6.13)	−2.69 (−10.59, 4.88)	**EA**					
5.63 (−0.54, 11.60)	5.06 (−2.99, 12.53)	7.73 (−2.41, 17.80)	**MOX**				
−1.46 (−5.92, 3.08)	−1.99 (−5.15, 0.88)	0.69 (−6.32, 7.88)	−7.06 (−14.51, 0.48)	**WM**			
−3.04 (−8.86, 2.65)	−3.57 (−11.31, 3.67)	−0.93 (−10.98, 9.19)	**−8.66 (−16.64, −0.38)** *	−1.55 (−8.94, 5.68)	**Placebo**		
**HAMD**							
**ACU**							
−2.35 (−8.20, 3.74)	**CH**						
0.40 (−4.13, 4.94)	2.73 (−3.85, 9.46)	**Combined therapies**					
−8.93 (−13.96, −2.94)	−6.65 (−14.00, 1.65)	−9.31 (−15.50, −2.50)	**EA**				
**11.66 (4.72, 18.38)** *	**13.99 (4.69, 23.20)** *	**11.22 (3.01, 19.16)** *	**20.58 (11.50, 28.94)** *	**MOX**			
−4.32 (−7.77, −0.76)	−1.93 (−7.85, 4.14)	−4.70 (−8.03, −1.37)	4.65 (−1.47, 10.03)	**−15.91 (−23.38, −8.11)** *	**WM**		
−4.34 (−11.13, 2.11)	−2.00 (−11.00, 6.73)	−4.79 (−12.81, 3.24)	4.70 (−4.54, 12.59)	**−15.99 (−25.30, −6.22)** *	−0.06 (−7.70, 7.21)	**Placebo**	
−5.48 (−22.89,11.99)	**CH**						
**Response rate**							
**ACU**							
**3.11 (1.34, 7.98)** *	**CH**						
1.24 (0.61, 2.62)	**0.39 (0.19, 0.80)** *	**Combined therapies**					
**4.37 (1.37, 15.47)** *	1.43 (0.34, 5.90)	3.60 (0.99, 13.49)	**EA**				
**0.29 (0.08, 0.93)** *	**0.09 (0.02, 0.36)** *	**0.23 (0.06, 0.85)** *	**0.06 (0.01, 0.33)**	**MOX**			
**6.13 (1.53, 28.73)** *	1.97 (0.45, 9.26)	**4.96 (1.30, 21.62)** *	1.38 (0.23, 9.01)	**22.16 (3.53, 148.10)** *	**WA**		
**4.48 (2.45, 8.65)** *	1.42 (0.71, 2.83)	**3.62 (2.35, 5.66)** *	1.02 (0.30, 3.43)	**15.59 (4.68, 61.21)** *	0.74 (0.18, 2.65)	**WM**	
2.81 (0.78, 11.02)	0.88 (0.19, 4.49)	2.31 (0.53, 10.61)	0.64 (0.10, 3.74)	**9.80 (2.90, 45.51)** *	0.46 (0.06, 3.09)	0.63 (0.15, 2.71)	**Placebo**

ACU, acupuncture; EA, electroacupuncture; MOX, moxibustion; CH, Chinese herb medicine; WA, warm acupuncture; combined therapies, the combination of acupuncture-related therapies and other therapies; WM, western medicine of anti-diarrheal or anti-spasmodic; placebo, and placebo acupuncture/blank control;*Significant difference. Bold values indicate significant differences.

As shown in Figure 3B, 12 studies reported SDS scores involving eight interventions and 923 patients. Here, we could find that after the consistency test, SDS scores included one closed loop, whose IF with 95% CI of these closed loops failed to contain 0, and the minimum values were 0.03 (Figure 4B). The results of the NMA showed that combined therapies were more efficacious than WM (SMD: −8.45; 95% CI: −15.50, −1.41; Table 4). The ranking results revealed that the top three interventions to reduce the SDS scores were combined therapies (92.1%), ACU (59.1%), and WA (55.4%), while the worst was WM (28.4%; Figure 5B).

As shown in Figure 3C, eight studies reported HAMA scores, involving six interventions and 551 patients. Inconsistency test results show that HAMA scores included one closed loop, whose IF with 95% CI of these closed loops contained 0, indicating no inconsistencies (Figure 4C). The results of the NMA showed that MOX was more efficacious than placebo (SMD: −8.66; 95% CI: −16.64, −0.38; Table 4). The ranking results depicted that the top three interventions to reduce the HAMA scores were MOX (99.7%), ACU (57.0%), and combined therapies (73.2%), while the worst was placebo (0%; Figure 5C).

As shown in Figure 3D, ten studies reported HAMD scores, involving seven interventions and 681 patients. Inconsistency test results show that HAMD scores included three closed loops, whose IF with 95% CI of 2 closed loops contained 0, indicating no obvious inconsistencies. In contrast, the IF with 95% CI of another one closed-loop failed to contain 0, and the minimum values were 0.03 (Figure 4D). The NMA results showed that MOX was more efficacious than the following interventions: ACU (SMD: 11.66; 95% CI: 4.72, 18.38), CH (SMD: 13.99; 95% CI: 4.69,23.20), combined therapies (SMD: 11.22; 95% CI: 3.01, 19.16), EA (SMD: 20.58; 95% CI: 11.50, 28.94), WM (SMD: −15.91; 95% CI: −23.38, −8.11), and placebo (SMD: −15.99; 95% CI: −25.30, −6.22). They all indicated that MOX could better relieve depression status for IBS-D patients (Table 4). The ranking results demonstrated that the top three interventions to reduce the HAMD scores were MOX (99.9%), combined therapies (73.7%), and ACU (68.8%), while the worst was placebo (9.9%; Figure 5D).

#### 3.4.2 Secondary outcome: Response rate

**FIGURE 6:**
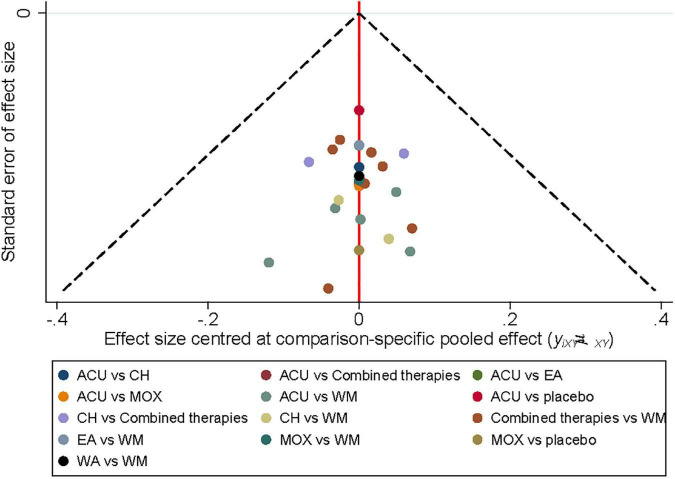
Funnel diagram. ACU, acupuncture; EA, electroacupuncture; MOX, moxibustion; CH, Chinese herb medicine; WA, warm acupuncture; combined therapies, the combination of acupuncture-related therapies and other therapies; WM, western medicine of anti-diarrheal or anti-spasmodic; placebo, and placebo acupuncture/blank control.

As shown in Figure 3E, 20 studies reported a response rate involving eight interventions and 1,639 patients. After the consistency test, the result of HAMD scores included seven closed loops, whose IF with 95% CI of six closed loops contained 0, indicating no obvious inconsistencies. In contrast, the IF with 95% CI of another one closed-loop failed to contain 0, and the minimum values were 0.01 (Figure 4E). The NMA results showed that MOX was more efficacious than the following interventions: ACU (SMD: 0.29; 95% CI:0.08,0.93), CH (SMD: 0.09; 95% CI: 0.02, 0.36), combined therapies (SMD: 0.23; 95% CI: 0.06, 0.85), EA (SMD: 0.06; 95% CI: 0.01, 0.33), WA (SMD: 22.16; 95% CI: 3.53, 148.10), WM (SMD: 15.59; 95% CI: 4.68, 61.21), and placebo (SMD: 9.80; 95% CI: 2.90, 45.51). Combined therapies was more efficacious than the following interventions: CH (SMD: 0.39; 95% CI: 0.19, 0.80), WA (SMD: 4.96; 95% CI: 1.30, 21.62), and WM (SMD: 3.62; 95% CI: 2.35, 5.66). ACU was more efficacious than the following interventions: CH (SMD: 3.11; 95% CI: 1.34, 7.98), EA (SMD: 4.37; 95% CI: 1.37, 15.47), WA (SMD: 6.13; 95% CI: 1.53, 28.73), and WM (SMD: 4.48; 95% CI: 2.45, 8.65; Table 4). They all indicated that acupuncture combined with other therapies improved the efficacy. The ranking results revealed that the top three interventions to reduce the HAMD scores were MOX (99.6%), ACU (79.7%), and combined therapies (75.8%), while the worst was WA (0.2%; Figure 5E). As shown by the funnel plot, all included studies were evenly distributed on either side of the vertical line *X* = 0, indicating that the likelihood of a small sample effect was quite low (Figure 6).

### 3.5 Adverse events

Among the 24 included trials, six reported adverse events occurred. Five trials with WM reported adverse outcomes like abdominal pain, dry stool, and nausea. One study with WM reported worsening diarrhea. Two studies with ACU as a treatment regimen reported subcutaneous bleeding and fainting after needling. Overall, studies using acupuncture as the primary intervention depicted a lower probability of adverse events than WM, indicating the safety of acupuncture-related therapies (Table 5).

**TABLE 5:** Adverse events.

References	Adverse events
Bu and Lv (55)	NR
Chen a et al. (56)	**WM:** 3 cases of dry and hard stool (3/30), 3 cases of headache (3/30)
Chen et al. (57)	NR
Han and Feng (58)	NR
Han et al. (59)	NR
Huang et al. (60)	NR
Li et al. (61)	NR
Li et al. (62)	**Combined therapies:** One case of abdominal pain (1/47) and one case of constipation (1/46) **WM:** One case of abdominal pain (1/47) and one case of constipation (1/46)
Meng (63)	**ACU:** 1 case of fainting after acupunctur (1/35) **WM:** 2 cases of rash (2/35), 1 case of pruritus (1/35), 2 cases of nausea (2/35)
Shu (64)	NR
Sun et al. (65)	NR
Tian et al. (66)	NR
Wang et al. (40)	NR
Yang et al. (41)	NR
Zhong a et al. (67)	NR
Zhou et al. (68)	NR
Zhong et al. (69)	NR
Chen (70)	**Combined therapies:** 1 case of fainting after acupuncture (1/34), 2 cases of subcutaneous hematoma (2/34) **WM:** 5 cases of dry stools (5/34), 4 cases of vomiting and regurgitation (4/34)
Li (71)	NR
Li (72)	**WM:** 1 case of worsening diarrhea (1/33)
Liang (73)	NR
Liu (74)	NR
Xiao (42)	NR
Yang (75)	**ACU:** 2 cases of subcutaneous hematoma (2/33)

## 4 Discussion

Our NMA included 24 RCTs involving 1,885 patients with IBS-D, evaluated eight interventions evaluated, and provided the following findings: (1) The most frequently used intervention, in addition to WM, was ACU, followed by combined therapies and EA; (2) The pairwise meta-analysis showed that ACU, combined therapies, and EA were all superior to WM in improving the anxiety and depression status of IBS-D patients. While the MNA results showed that MOX, ACU, combined therapies, and EA were all superior to WM. Overall, MOX, ACU, and combined therapies had the highest benefit; (3) The ranking results revealed that MOX, ACU, combined therapies, and EA all had high SUCRA rankings for different outcome indicators, indicating that these interventions could serve as optimal interventions to improve anxiety and depression status in patients with IBS-D; (4) The results of adverse events depicted that the rate of adverse events was lower with acupuncture-related therapy than with drugs, establishing the safety of acupuncture-related therapy over pharmacotherapy.

The NMA method utilized in this study can compensate for the lack of direct data by using indirect data which compares the efficacy of different acupuncture-related therapies against anxiety and depression status in patients with IBS-D. The results provide some evidence for the clinical efficacy of acupuncture-related treatments in the treatment of IBS-D. Compared with previous meta-analysis (17), our study used an NMA to comprehensively evaluate the included literature, rank the different interventions, and calculate the best probability. Moreover, our results also confirmed the potential of acupuncture-related therapies for IBS-D. Meanwhile, we focused on the anxiety and depression status of IBS-D patients and selected suitable outcome indicators to evaluate the improvement of acupuncture-related therapies.

IBS occurrence involves many factors, including altered gastrointestinal dynamics (43), abnormal visceral sensation (44), abnormal brain-gut axis interaction (45), intestinal inflammation (46), and micro-ecological imbalance (47). At the same time, abnormal psychiatric symptoms like anxiety and depression are also important causes of its development (48). The severity of IBS bowel symptoms is prominently related to anxiety or depression problems (45, 49, 50), and it can also harm the quality of life and treatment efficacy of patients (51). In this study, the SAS and HAMA scores were used to evaluate the anxiety of patients; the SDS and HAMD scores were used to evaluate the depression of patients. However, SAS and SDS were self-assessment scales, which can intuitively reflect the patient’s subjective feelings; while HAMA and HAMD were other-assessment scales, with more detailed descriptions of each item, more delicate assessment, and more objective results. As a characteristic therapy of Traditional Chinese Medicine (TCM), acupuncture has good efficacy in many diseases and has a broad spectrum of diseases. Various literature reports on acupuncture as the primary intervention for IBS have depicted beneficial effects on improving IBS symptoms. In recent years, increasing studies have confirmed the efficacy of acupuncture for various types of depression, and these studies suggest that acupuncture has great potential for the treatment of anxiety and depression (52–54). It improves a possible mechanism for acupuncture therapy in IBS.

Nevertheless, our study has certain limitations: (1) most of the included studies were published in Chinese journals, creating a publication bias involving the Chinese culture; (2) The risk of bias assessment of the included studies was mostly unclear, and the quality of the literature was low. Therefore, in future studies, reporting should be based on the Consolidated Standards of Reporting Trials (CONSORT) to ascertain the literature quality; (3) Since acupuncture operations cannot be blinded to patients, the principle of triple separation of clinical operators, efficacy evaluators, and statistical analysts can be utilized, in addition to reporting the qualifications of acupuncturists and acupuncture responses in clinical trials as required by the Standards for Reporting Interventions in Clinical Trials of Acupuncture (STRICTA) statement; (4) Due to the limitations within the original literature, for combining two therapies (one of which is acupuncture related therapy), we uniformly grouped them into combined treatments, including ACU + MOX, ACU + WM, ACU + CH three types. The results suggest that combined therapies have better performance in improving the leading outcome indicators. However, there are many combinations of combined therapies, which will be further elaborated in subsequent studies; (5) Chinese herbal medicine has also been reported to be a more common and effective therapy for both symptom relief of IBS-D and the improvement of anxiety and depressive status. In order to comprehensively include the published studies using acupuncture-related therapies for IBS-D, we have retained the studies comparing acupuncture-related therapies with Chinese herbal medicine. In our results, Chinese herbal medicine also showed some effect, however, because this study focused on acupuncture-related therapies, the literature using Chinese herbal medicine was not included comprehensively, so the results of Chinese herbal medicine may be biased, and we will comprehensively evaluate Chinese herbal medicine in our follow-up study; (6) the literature included in this study was focused on short-term efficacy, and the long-term effectiveness of acupuncture-related therapies for IBS-D remains to be determined.

## 5 Conclusion

Among the included interventions, MOX, ACU, combined therapies, and EA could be the optimal therapies to improve the anxiety and depression status of patients with IBS-D. In addition, combining acupuncture-related therapies with other therapies has a high overall benefit and safety, and we recommend using these therapies in clinical settings.

## Data availability statement

The original contributions presented in this study are included in the article/Supplementary material, further inquiries can be directed to the corresponding author.

## Author contributions

XW, CL, and YS conceived this study. XW drafted the manuscript. XW and YS revised the study design. XS, JL, and JZ performed the study search, study selection, and collected the data. YH, GZ, and GL evaluated the quality of the included study. XW, GZ, and YS conducted the data analysis. CL and YS reviewed and revised the manuscript. All authors have read and approved the publication.

## Footnotes

1
https://www.crd.york.ac.uk/PROSPERO

## Supplementary material

Supplementary Appendix 1 The PRISMA-NMA checklist for the included studies used in the NMA.

Supplementary Appendix 2 The search strategies for the included RCTs for each database.

## References

[1] Lacy B Mearin F Chang L Chey W Lembo A Simren M Bowel disorders. *Gastroenterology.* (2016) 150:1393–407. 10.1016/s0016-5085(15)32239-3 27144627

[2] Lovell R Ford A. Global prevalence of and risk factors for irritable bowel syndrome: a meta-analysis. *Clin Gastroenterol Hepatol.* (2012) 10:712–21.e4. 10.1016/j.cgh.2012.02.029 22426087

[3] Lacy B. Emerging treatments in neurogastroenterology: eluxadoline–a new therapeutic option for diarrhea-predominant IBS. *Neurogastroenterol Motil.* (2016) 28:26–35. 10.1111/nmo.12716 26690872

[4] Li X Chang M Xu D. The current status of the epidemiological study of irritable bowel syndrome in China. *Chin J Gastroenterol Hepatol.* (2013) 22:734–9. 10.3969/j.issn.1006-5709.2013.08.005

[5] Canavan C West J Card T. The epidemiology of irritable bowel syndrome. *Clin Epidemiol.* (2014) 06:71–80. 10.1016/s0300-5089(21)00724-0 PMC3921083 24523597

[6] Long Y Huang Z Deng Y Chu H Zheng X Yang J Prevalence and risk factors for functional bowel disorders in South China: a population based study using the Rome III criteria. *Neurogastroenterol Motil.* (2017) 29:e12897. 10.1111/nmo.12897 27412422

[7] Sperber A Dumitrascu D Fukudo S Gerson C Ghoshal U Gwee K The global prevalence of IBS in adults remains elusive due to the heterogeneity of studies: a Rome foundation working team literature review. *Gut.* (2017) 66:1075–82. 10.1136/gutjnl-2015-311240 26818616

[8] Creed F. The incidence and risk factors for irritable bowel syndrome in population-based studies. *Aliment Pharmacol Ther.* (2019) 50:507–16. 10.1111/apt.15396 31313850

[9] Brandt L Chey W Foxx-Orenstein A Schiller L Schoenfeld P Spiegel B An evidence-based position statement on the management of irritable bowel syndrome. *Am J Gastroenterol.* (2009) 104:S1–35. 10.1038/ajg.2008.122 19521341

[10] Kabra N Nadkarni A. Prevalence of depression and anxiety in irritable bowel syndrome: a clinic based study from India. *Indian J Psychiatry.* (2013) 55:77–80. 10.4103/0019-5545.105520 23439939 PMC3574461

[11] Chitkara D van Tilburg M Blois-Martin N Whitehead W. Early life risk factors that contribute to irritable bowel syndrome in adults: a systematic review. *Am J Gastroenterol.* (2008) 103:765–74. 10.1111/j.1572-0241.2007.01722.x 18177446 PMC3856200

[12] Drossman D. Functional gastrointestinal disorders: history, pathophysiology, clinical features, and Rome IV. *Gastroenterology.* (2016) 150:1262–79. 10.1053/j.gastro.2016.02.032 27144617

[13] Camilleri M Boeckxstaens G. Dietary and pharmacological treatment of abdominal pain in IBS. *Gut.* (2017) 66:966–74. 10.1136/gutjnl-2016-313425 28232472

[14] Wang Z Xu M Shi Z Bao C Liu H Zhou C Mild moxibustion for Irritable Bowel Syndrome with Diarrhea (IBS-D): a randomized controlled trial. *J Ethnopharmacol.* (2022) 289:115064. 10.1016/j.jep.2022.115064 35114338

[15] Bao C Wu L Shi Y Shi Z Jin X Shen J Long-term effect of moxibustion on irritable bowel syndrome with diarrhea: a randomized clinical trial. *Ther Adv Gastroenterol.* (2022) 15:17562848221075131. 10.1177/17562848221075131 35222693 PMC8874177

[16] Zheng H Li Y Zhang W Zeng F Zhou S Zheng H Electroacupuncture for patients with diarrhea-predominant irritable bowel syndrome or functional diarrhea: a randomized controlled trial. *Medicine.* (2016) 95:e3884. 10.1155/2022/2564979 27310980 PMC4998466

[17] Guo J Xing X Wu J Zhang H Yun Y Qin Z Acupuncture for adults with diarrhea-predominant irritable bowel syndrome or functional diarrhea: a systematic review and meta-analysis. *Neural Plast.* (2020) 2020:8892184. 10.1155/2020/8892184 33299403 PMC7705439

[18] Zhu L Ma Y Ye S Shu Z. Acupuncture for diarrhoea-predominant irritable bowel syndrome: a network meta-analysis. *Evid Based Complement Alternat Med.* (2018) 2018:2890465. 10.1155/2018/2890465 29977312 PMC5994265

[19] Wang X Li J Wang Y Yu C He C Huang Z Acupuncture and related therapies for the cognitive function of Alzheimer’s disease: a network meta-analysis. *Iran J Public Health.* (2021) 50:2411–26. 10.18502/ijph.v50i12.7924 36317033 PMC9577142

[20] Lim B Manheimer E Lao L Ziea E Wisniewski J Liu J Acupuncture for treatment of irritable bowel syndrome. *Cochrane Database Syst Rev.* (2006) 5:Cd005111. 10.1002/14651858.CD005111 17054239

[21] Manheimer E Wieland L Cheng K Li S Shen X Berman B Acupuncture for irritable bowel syndrome: systematic review and meta-analysis. *Am J Gastroenterol.* (2012) 107:835–47. 10.1038/ajg.2012.66 22488079 PMC3671917

[22] Hutton B Salanti G Caldwell D Chaimani A Schmid C Cameron C The PRISMA extension statement for reporting of systematic reviews incorporating network meta-analyses of health care interventions: checklist and explanations. *Ann Int Med.* (2015) 162:777–84. 10.7326/m14-2385 26030634

[23] Wang X Chen Y Liu Y Yao L Estill J Bian Z Reporting items for systematic reviews and meta-analyses of acupuncture: the PRISMA for acupuncture checklist. *BMC Complement Altern Med.* (2019) 19:208. 10.1186/s12906-019-2624-3 31405367 PMC6689876

[24] Wang X Shi X Lv J Zhang J Huo Y Zuo G *Acupuncture and Related Therapies for Anxiety and Depression in Diarrhoea-Predominant Irritable Bowel Syndrome(IBS-D): A Protocol for Systematic Review and Network Meta-Analysis of Randomized Controlled Trials PROSPERO.* (2022). Available online at: https://www.crd.york.ac.uk/prospero/display_record.php?ID=CRD42022364560 (accessed November 22, 2022). 10.3389/fpsyt.2022.1067329 PMC9816906 36620677

[25] Badia X Mearin F Balboa A Baró E Caldwell E Cucala M Burden of illness in irritable bowel syndrome comparing Rome I and Rome II criteria. *Pharmacoeconomics.* (2002) 20:749–58. 10.2165/00019053-200220110-00004 12201794

[26] Markert C Suarez-Hitz K Ehlert U Nater U. Endocrine dysregulation in women with irritable bowel syndrome according to Rome II criteria. *J Behav Med.* (2016) 39:519–26. 10.1007/s10865-016-9718-x 26846219

[27] Drossman D Dumitrascu D. Rome III: new standard for functional gastrointestinal disorders. *J Gastrointestin Liver Dis.* (2006) 15:237–41. 17013448

[28] Barberio B Houghton L Yiannakou Y Savarino E Black C Ford A. Symptom stability in Rome IV vs Rome III irritable bowel syndrome. *Am J Gastroenterol.* (2021) 116:362–71. 10.14309/ajg.0000000000000946 33009062

[29] Study Group of Functional Gastmointestinal Disorders, Study Group of Gastrointestinal Motility, Chinese Society of Gastroenterology, Chinese Medical Association. Chinese expert consensus of imitable bowel syndrome in 2020. *Chin J Dig.* (2020) 40:803–18. 10.3760/cma.j.cn311367-20201116-00660 30704229

[30] Jie M Ying L Lai-ping Z Chen-ping Z Zhi-yuan Z. Comparison between Jadad scale and Cochrane collaboration’s tool for assessing risk of bias on the quality and risk of bias evaluation in randomized controlled trials. *China J Oral Maxillofac Surg.* (2012) 10:417–22. 10.1007/s00402-021-04326-9 35024906

[31] Higgins J Green S. *Cochrane Handbook for Systematic Reviews of Interventions: The Cochrane Collaboration.* (2008). Available online at: http://www.cochrane.org/training/cochrane-handbook (accessed June 18, 2022).

[32] Dan W Junxia Z Zhenyun M Hongxia Z Xiaodong Z Xueyi W Discussing on the research of heterogeneity in meta-analysis. *Chin J Evid Based Med.* (2009) 9:1115–8.

[33] Shim S Yoon B Shin I Bae J. Network meta-analysis: application and practice using Stata. *Epidemiol Health.* (2017) 39:e2017047. 10.4178/epih.e2017047 29092392 PMC5733388

[34] Higgins J Thompson S Deeks J Altman D. Measuring inconsistency in meta-analyses. *BMJ.* (2003) 327:557–60. 10.1136/bmj.327.7414.557 12958120 PMC192859

[35] Wen J Li Y. The selection of a summary statistic for use in meta-analysis. *Chin J Evid Based Med.* (2007) 7:606–13. 10.3969/j.issn.1672-2531.2007.08.014

[36] Zhang C Yan J Sun F Liu Q Guo Y Zeng X. Differentiation and handling of homogeneity in network meta-analysis. *Chin J Evid Based Med.* (2014) 14:884–8. 10.7507/1672-2531.20140146

[37] Liu L Zhang M Li J. Bayesian econometrics based on WinBUGS. *J East China Univ Technol.* (2007) 26:101–7. 10.3969/j.issn.1674-3512.2007.02.001

[38] Salanti G Ades A Ioannidis J. Graphical methods and numerical summaries for presenting results from multiple-treatment meta-analysis: an overview and tutorial. *J Clin Epidemiol.* (2011) 64:163–71. 10.1016/j.jclinepi.2010.03.016 20688472

[39] Chaimani A Higgins J Mavridis D Spyridonos P Salanti G. Graphical tools for network meta-analysis in STATA. *PLoS One.* (2013) 8:e76654. 10.1371/journal.pone.0076654 24098547 PMC3789683

[40] Wang S Wang X Yang R Xu Y Li M. Efficacy and mechanism of acupuncture combined with Tongxieyaofang for diarrhea-type irritable bowel syndrome of liver depression and Spleen deficiency. *Chin Acupunct Moxibustion.* (2020) 40:605–9. 10.13703/j.0255-2930.20190818-k0004 32538010

[41] Yang M Zou R Zhang L Xu P. Clinical study of acupuncture on the psycho-psychological status of patients with irritable bowel syndrome of liver depression and Spleen deficiency Hubei. *J Tradit Chin Med.* (2020) 42:51–4.

[42] Xiao J. *Clinical Application of Five Elements Acupuncture and Acupuncture in the Treatment of Spleen and Kidney Yang Deficiency Diarrhea Type Irritable Bowel Syndrome*. Master’s thesis. Guangxi: Guangxi University of Chinese Medicine (2021).

[43] Camilleri M McKinzie S Busciglio I Low P Sweetser S Burton D Prospective study of motor, sensory, psychologic, and autonomic functions in patients with irritable bowel syndrome. *Clin Gastroenterol Hepatol.* (2008) 7:772–81. 10.1016/j.cgh.2008.02.060 18456567 PMC2495078

[44] Greenwood-Van Meerveld B Prusator D Johnson A. Animal models of gastrointestinal and liver diseases. Animal models of visceral pain: pathophysiology, translational relevance, and challenges. *Am J Physiol Gastrointest Liver Physiol.* (2015) 11:G885–903. 10.1152/ajpgi.00463.2014 25767262

[45] Koloski N Jones M Kalantar J Weltman M Zaguirre J Talley N. The brain–gut pathway in functional gastrointestinal disorders is bidirectional: a 12-year prospective population-based study. *Gut.* (2012) 61:1284–90. 10.1136/gutjnl-2011-300474 22234979

[46] Ohman L Simrén M. Pathogenesis of IBS: role of inflammation, immunity and neuroimmune interactions. *Nat Rev Gastroenterol Hepatol.* (2010) 3:163–73. 10.1038/nrgastro.2010.4 20101257

[47] Carroll I Ringel-Kulka T Keku T Chang Y Packey C Sartor R Molecular analysis of the luminal- and mucosal-associated intestinal microbiota in diarrhea-predominant irritable bowel syndrome. *Am J Physiol Gastrointest Liver Physiol.* (2011) 5:G799–807. 10.1152/ajpgi.00154.2011 21737778 PMC3220325

[48] Qi L Li H Yang N Li Y Tan C Yang J Mechanism research and clinical progress of acupuncture for irritable bowel syndrome. *Chin Acupunct Moxibustion.* (2022) 42:231–6. 10.13703/j.0255-2930.20210131-0003 35152593

[49] Fond G Loundou A Hamdani N Boukouaci W Dargel A Oliveira J Anxiety and depression comorbidities in irritable bowel syndrome (IBS): a systematic review and meta-analysis. *Eur Arch Psychiatry Clin Neurosci.* (2014) 264:651–60. 10.1007/s00406-014-0502-z 24705634

[50] Modabbernia M Mansour-Ghanaei F Imani A Mirsafa-Moghaddam S Sedigh-Rahimabadi M Yousefi-Mashhour M Anxiety-depressive disorders among irritable bowel syndrome patients in Guilan, Iran. *BMC Res Notes.* (2012) 5:112. 10.1186/1756-0500-5-112 22353390 PMC3392738

[51] Zamani M Alizadeh-Tabari S Zamani V. Systematic review with meta-analysis: the prevalence of anxiety and depression in patients with irritable bowel syndrome. *Aliment Pharmacol Ther.* (2019) 50:132–43. 10.1111/apt.15325 31157418

[52] Zhang Z Chen H Yip K Ng R Wong V. The effectiveness and safety of acupuncture therapy in depressive disorders: systematic review and meta-analysis. *J Affect Disord.* (2010) 124:9–21. 10.1016/j.jad.2009.07.005 19632725

[53] Wong Y Wu J Zhou G Zhu F Zhang Q Yang X Antidepressant monotherapy and combination therapy with acupuncture in depressed patients: a resting-state functional near-infrared spectroscopy (fNIRS) study. *Neurotherapeutics.* (2021) 18:2651–63. 10.1007/s13311-021-01098-3 34431029 PMC8804104

[54] Armour M Smith C Wang L Naidoo D Yang G MacPherson H Acupuncture for depression: a systematic review and meta-analysis. *J Clin Med.* (2019) 8:1140. 10.3390/jcm8081140 31370200 PMC6722678

[55] Bu L Lv D. Effect of “ShuGanJianPi” acupuncture for brain and intestinal peptides and anxiety and depression in patients with Diarrhoea-Predominant Irritable Bowel Syndrome. *Mod J Integr Tradit Chin West Med.* (2020) 29:1074–7. 10.3969/j.issn.1008-8849.2020.10.012

[56] Chen Q Zhou Y Zhang M Xie J Miao H Gong Y. Efficacy observation of acupuncture combined with salt-partitioned moxibustion for IBS-D of Spleen deficiency pattern. *Shanghai J Acupunct Moxibustion.* (2021) 40:400–5. 10.13460/j.issn.1005-0957.2020.13.1082

[57] Chen Y Chen X Yin X Shi Y. Comparison of the therapeutic effects of electroacupuncture and pmbiotics combined with deanxit in treating diarrhea-predominant irritable bowel syndrome. *Mod J Integr Tradit Chin West Med.* (2012) 32:594–8. 22679715

[58] Han B Feng L. Effect of combined drug-acupuncture treatment on cytokines in patients with diarrhoea-predominant irritable bowel syndrome in anxiety state. *Chin J Clin Rational Drug Use.* (2013) 6:121–2. 10.15887/j.cnki.13-1389/r.2013.14.087

[59] Han Z Ren L Lu F Ruan C Tang B. Analysis of the acupuncture combined with traditional chinese medicine in the treatment of diarrhea-predominant pattern irritable bowel syndrome patients. *Chin J Gen Pract.* (2019) 17:1911–3. 10.16766/j.cnki.issn.1674-4152.001088

[60] Huang H Fang F Zhai Y Li L Song F. Clinical Study on du-meridian moxibustion for diarrhea-predominant irritable bowel syndrome. *New Chin Med.* (2019) 51:241–3. 10.13457/j.cnki.jncm.2019.09.072

[61] Li H Zhou Y Li Z Zhu L Xiong J Li Y Clinical observation on umbilical moxibustion therapy treating 30 cases of diarrhea type irritable bowel syndrome with stagnation of liver Qi and Spleen deficiency. *J Tradit Chin Med.* (2018) 59:2034–6. 10.13288/j.11-2166/r.2018.23.014

[62] Li Y Xu P Yang M Pan X. Therapeutic observation of acupuncture-moxibustion for diarrhea-dominant irritable bowel syndrome of liver depression and Spleen deficiency pattern. *Shanghai J Acupunct Moxibustion.* (2021) 40:1305–11. 10.13460/j.issn.1005-0957.2021.13.0032

[63] Meng G. Acupuncture treatment for depressive symptom in diarrhea-predominant irritable bowel syndrome:a randomized controlled study. *J Acupunct Tuina Sci.* (2019) 17:422–6. 10.1007/s11726-019-1138-3

[64] Shu Y. Effect of warm acupuncture combined with acupuncture treatment on patients’ defecation and mood in diarrhoea-predominant irritable bowel syndrome. *J Taishan Med Coll.* (2018) 39:785–7. 10.3969/j.issn.1004-7115.2018.07.019

[65] Sun Y Wang S Yu T. Tiaoshen acupuncture method combined with electroacupuncture for diarrhea-type irritable bowel syndrome : a randomized controlled trial. *Chin Acupunct Moxibustion.* (2021) 41:13–6. 10.13703/j.0255-2930.20191220-k0001 33559435

[66] Tian W Wei J Li Q You W Li D Zhang Y. Therapeutic effect of old ten needling combined with warming-dredging acupuncture on irritable bowel syndrome caused by emotional stress disorder after stroke. *Liaoning J Tradit Chin Med.* (2020) 47:162–5. 10.13192/j.issn.1000-1719.2020.08.047

[67] Zhong F Cao Y Luo R Sheng R Shi W Liu Y Clinical effect of electroacupuncture at quchi and shangjuxu in treatment of diarrhea-predominant irritable bowel syndrome. *J Anhui Univ Chin Med.* (2018) 37:68–71. 10.3969/j.issn.2095-7246.2018.02.020

[68] Zhou P Zeng Z Jiang Q Su Y Ye X. Clinical research on combined treatment with modified xiao-yao powder and acupuncture for diarrhea-predominant irritable bowel syndrome. *World Sci Technol.* (2014) 16:1331–5. 10.11842/wst.2014.06.023

[69] Zhong F Cao Y Luo R Sheng R Shi W Liu Y Clinical effect of electroacupuncture at he-mu acupoint combination in treatment of diarrhea-predominant irritable bowel syndrome. *J Hunan Univ Chin Med.* (2018) 38:435–8. 10.3969/j.issn.1674-070X.2018.04.018

[70] Chen Q. *Clinical Study on the Treatment of Diarrhea-predominant Irritable Bowel Syndrome with Mind-regulating Acupuncture Combined with Medicine-Separated.* Master’s thesis. Yunnan: Yunnan University of Chinese Medicine (2021).

[71] Li H. *Clinical Efficacy of “ShuGanJianPi” Acupuncture for Diarrhoea-Predominant Irritable Bowel Syndrome Patients.* Master’s thesis. Nanjing: Nanjing University of Chinese Medicine (2011).

[72] Li J. *Clinical Efficacy Evaluation of Acupuncture with Regulating Mind and Spleen for Diarrhea-predominant Irritable Bowel Syndrome and its Brain Functional Changes in Rs-fMRI.* Doctoral thesis. Nanjing: Nanjing University of Chinese Medicine (2017).

[73] Liang S. *Effect of Acupuncture with Regulating Mind And Strengthening Spleen on Hypothalamus-Pituitary-Adrenal Axis in Patients with Diarrhea-predominant Irritable Bowel Syndrome*. Master’s thesis. Nanjing: Nanjing University of Chinese Medicine (2017).

[74] Liu F. *The Clinical Observation on the Severity and Mental state of Patients with Diarrhea Type Irritable Bowel Syndrome of stagnation of liver-qi and deficiency of the spleen Cured with Herb-partitioned moxibustion on the Navel*. Master’s thesis. Shandong: Shandong University of Traditional Chinese Medicine (2017).

[75] Yang T. *The Clinical Curative Effect Observation of Acupuncture “Shenque Eight Array” on the Treatment of Irritable Bowel Syndrome with Diarrhea of Spleen Asthenia and Damp Abundance*. Master’s thesis. Sichuan: Chengdu University of Traditional Chinese Medicine (2020).

